# Evaluation of Lung and Bronchoalveolar Lavage Fluid Oxidative Stress Indices for Assessing the Preventing Effects of Safranal on Respiratory Distress in Diabetic Rats

**DOI:** 10.1155/2014/251378

**Published:** 2014-02-20

**Authors:** Saeed Samarghandian, Reza Afshari, Aghdas Sadati

**Affiliations:** ^1^Department of Basic Medical Sciences, Neyshabur Medical Faculty of Sciences, Neyshabur, Iran; ^2^Health Strategic Research Center, Neyshabur Medical Faculty of Sciences, Neyshabur, Iran; ^3^Addiction Research Centre, Mashhad University of Medical Sciences, Mashhad, Iran

## Abstract

We investigated the effects of antioxidant activity of safranal, a constituent of *Crocus sativus* L., against lung oxidative damage in diabetic rats. The rats were divided into the following groups of 8 animals each: control, diabetic, and three diabetic + safranal-treated (0.25, 0.50, and 0.75 mg/kg/day) groups. Streptozotocin (STZ) was injected intraperitoneally (i.p.) at a single dose of 60 mg/kg for diabetes induction. Safranal was administered (i.p.) from 3 days after STZ administration to the end of the study. At the end of the 4-week period, malondialdehyde (MDA), nitric oxide (NO) and reduced glutathione (GSH) contents, activity of superoxide dismutase (SOD), and catalase (CAT) were measured in the bronchoalveolar lavage fluid (BALF) and lung tissue. Safranal in the diabetic groups inhibited the level of MDA and NO in BALF supernatant and lung homogenate. The median effective dose (ED_50_) values were 0.42, 0.58, and 0.48, 0.71 mg/kg, respectively. Safranal in the diabetic groups increased the level of GSH and the activity of CAT and SOD in BALF supernatant and lung homogenate. The ED_50_ values were 0.25, 0.33, 0.26 in BALF and 0.33, 0.35, 0.46 mg/kg in lung, respectively. Thus, safranal may be effective to prevent lung distress by amelioration oxidative damage in STZ diabetic rats.

## 1. Introduction

Oxidative stress has been implicated in the major complications of diabetes mellitus (DM), including retinopathy, nephropathy, neuropathy, and accelerated coronary artery disease. Recently, several epidemiological and experimental studies have been reported that DM is an independent risk factor for occurrence respiratory disorders such as asthma [1]. Hyperglycemia due to DM leads to increasing oxidative stress and inflammatory responses [2]. Oxidative stress and inflammatory mediators are responsible mechanisms for induction of the pulmonary distress. The combination of these mechanisms alters the production of the oxidants, causing cellular stress and consequently the structural damage [3]. The complications of diabetes mellitus are the main causes of morbidities and mortalities [4]. However, antidiabetic drugs can not prevent diabetes complications significantly [5]. Therefore, this is necessary to provide drugs with lesser adverse effects and greater benefit to control diabetes and its complications [5]. Nowadays, with attention this issue, ethnobotanical studies that have focused on the protective effects of natural antioxidants have been originated from plants directly or indirectly [6]. Saffron (dried stigmas of *Crocus sativus* L.) is a food additive that used in the traditional medicine for the treatment of numerous diseases including depression, cognitive disorders, seizures, and cancer [7, 8]. Scientific findings have showed that saffron and the important ingredients (safranal and crocin) have antitumor, antigenotoxic, memory and learning enhancing, neuroprotective, analgesic, and anti-inflammatory, anticonvulsant, antianxiety, anti-depressant, antihypertensive and antihyperlipidemic effects [7–10]. Recently, it was reported that the saffron extract, crocin, and safranal exhibited significant radical scavenging activity and thus antioxidant activity [11, 12].

Considering the antioxidant effects of safranal, this study was designed to evaluate the protective activity of safranal against pulmonary damage due to oxidative stress in streptozotocin- (STZ-) diabetic rats.

## 2. Materials and Methods

### 2.1. Chemicals and Reagents

Streptozotocin and safranal were from Sigma (St. Louis, MO, USA). All other chemicals used were of analytical grade and were obtained from Merck.

### 2.2. Animals

45 male rats (2 months; 200 ± 13 g) were bred at the university experimental animal care centre. Animals were maintained under standard environmental conditions and had free access to standard rodent feed and water.

### 2.3. Study Design

45 male Wistar albino rats were randomly allotted into five experimental groups, as follows: group 1, control (C; *n* = 8); group 2, diabetic (D; *n* = 8); group 3, diabetic+safranal-treated (0.25 mg/kg/day) (D + S1; *n* = 8); group 4, diabetic + safranal-treated (0.5 mg/kg/day) (D + S2; *n* = 8); and group 5, diabetic+safranal-treated (0.75 mg/kg/day) (D + S3; *n* = 8). Rats were kept in their own cages at constant room temperature (21 ± 2°) under a normal 12 hr light: 12 hr dark cycle with free access to food and water. The animals were housed according to regulation of the Walfare of experimented animals. The study was conducted in Mashhad Medical University Experimental Animal Research Laboratory. Protocols were approved by the Ethical Committee. On the first day of the study, the all above diabetic groups were given STZ in a single intraperitoneal (i.p.) injection at a dose of 60 mg/kg for induction of diabetes. Blood was extracted from the tail vein for glucose analysis 72 hours after streptozotocin injection. The rats with blood glucose levels higher than 250 mg/dL were accepted as diabetic. In the control groups (C), safranal vehicle (i.p.) was administered to the treatment groups from 3 days after STZ administration; the injection continued to the end of the study (for 4 weeks). Blood glucose level and body weights were recorded at weekly intervals. The animals were sacrificed under light anesthesia (diethyl ether) 1 day after the end of the treatment, at which time blood was collected from retro-orbital sinus. Trachea and lungs were removed immediately for preparation lung lavage and lung homogenate.

### 2.4. Preparation Lung Lavage

Lung lavage was performed by cannulating the trachea and instilling 8.0 mL of cold normal saline with a syringe. The lavage fluid was rinsed in and out three times before collection [13]. The sample was centrifuged (2000 g, 5 min, 4°C) and the supernatant frozen at −70°C until being assayed.

### 2.5. Preparation Lung Homogenate

Lung homogenate was obtained from the right lung. The tissue was homogenized with KCl in 1 : 10 ratio. The homogenate was centrifuged (9000 ×g, 30 min) and the supernatant was used for measurement of oxidative stress indices.

### 2.6. Measurement of Reduced Glutathione (GSH)

GSH was determined by the method of Ellman (1959). We added 10% of trichloroacetic acid (TCA) to the homogenate then, centrifuged. 1.0 mL of supernatant was treated with 0.5 mL of Ellman's reagent (19.8 mg of 5,5-dithiobisnitrobenzoic acid (DTNB) in 100 mL of 0.1% sodium nitrate) and 3.0 mL of phosphate buffer (0.2 M, pH 8.0). The absorbance was read at 412 nm [14].

### 2.7. Measurement of Malondialdehyde (MDA)

MDA levels, as an index of lipid peroxidation, were measured in the homogenate. MDA reacts with thiobarbituric acid (TBA) as a thiobarbituric acid reactive substance (TBARS) to produce a red colored complex which has peak absorbance at 532 nm. Three mL phosphoric acid (1%) and 1 ml TBA (0.6%) were added to 0.5 mL of liver homogenate in a centrifuge tube and the mixture was heated for 45 min in a boiling water bath. After cooling butanol was added the mixture and vortex-mixed for 1 min followed by centrifugation at 20000 rpm for 20 min. The organic layer was transferred to a fresh tube and its absorbance was measured at 532 nm and compared with values obtained from MDA standards. Results are expressed as nmol/mg tissue [15].

### 2.8. Catalase (CAT)

CAT was assayed colorimetrically at 620 nm and expressed as moles of H_2_O_2_ consumed/min/mg protein as described by Sinha, (1972). The reaction mixture contained phosphate buffer (0.01 M, pH 7.0), tissue homogenate and 2 M H_2_O_2_. The reaction was stopped by the addition of dichromate acetic acid reagents (5% potassium dichromate and glacial acetic acid were mixed in a ratio of 1 : 3), [16, 17].

### 2.9. Measurement of Superoxide Dismutase (SOD) Activity

SOD was measured based on inhibition of the formation of amino blue tetrazolium formazan in nicotinamide adenine dinucleotide, phenazine methosulfate and nitroblue tetrazolium (NADH-PMS-NBT) system, according to method of Kakkar et al. (1984). One unit of enzyme activity was expressed as 50% inhibition of NBT reduction [18].

### 2.10. Measurement of Nitric Oxide (NO)

NO level can be determined spectrophotometrically by measuring the accumulation of its stable degradation products, nitrite and nitrate. The serum nitrite level was determined by the Griess reagent according to Hortelano et al., (1995). The Griess reagent, a mixture (1 : 1) of 1% sulfanilamide in 5% phosphoric acid and 0.1% 1-naphtyl ethylenediamine gives a red-violent diazo color in the presence of nitrite. The color intensity was measured at 540 nm. Results were expressed as *μ*mol/l using a NaNO2 calibration graph [19].

### 2.11. Measurement of Protein Content

Protein content was determined by the method of Lowry and coworkers 1951, using bovine serum albumin (BSA) as a standard [20].

### 2.12. Statistical Analysis

The data were expressed as means ± SEM. Statistical analysis was performed by SPSS/16 statistical software for Microsoft Windows, (Professional Statistic). Data were analyzed using one-way analysis of variance (ANOVA). The homogeneity of variance was tested by use of the Levene test. The Tukey honestly significant difference test was used for post hoc wise analysis of the data with homogenous variances, whereas Tamhane's post hoc pair wise analysis of data was used for data sets with nonhomogenous variances. Statistically significant differences in results of morphometric quantifications were determined by the Student's t-test. A 2-tailed P value less than 0.05 was considered statistically significant. Statistical analysis of the results concerning SOD, NO, GSH, and MDA levels were performed by one-way ANOVA followed by the Bonferroni post hoc test for multiple comparisons.

## 3. Result

STZ injection produced significant oxidative stress in the BALF and lung homogenate of diabetic rats 4 weeks after DM induction which was manifested by increased lipid peroxidation products (MDA) and with decreased GSH compared to control group (*P* < 0.001), (Tables 1 and 2).

Safranal treatment significantly decreased the MDA in the BALF and lung homogenate and also increased in the glutathione in diabetic safranal (0.25, 0.5 and 0.75 mg/kg/day)—treated groups versus the nontreated diabetic group (*P* < 0.001). However, the effect of the lowest concentration of safranal on MDA level in BALF was with similar value as in nontreated diabetic rats. The activity of GSH in BALF of animals has received the high safranal concentration were significantly greater than the low concentration (*P* < 0.01) (Table 1). Our results also showed that GSH activity in lung homogenate of diabetic rats treated with the high concentration was also significantly increased compared with the low (*P* < 0.001) and middle concentrations (*P* < 0.05), (Table 2). Safranal in the diabetic groups increased the level of GSH in BALF supernatant and lung homogenate. The ED_50_ values were 0.25 and 0.33 mg/kg, respectively. In addition, the levels of MDA in BALF of animals that have been administrated the high safranal concentration were significantly lower than the low (*P* < 0.05) and middle concentrations (*P* < 0.01) (Table 1); however, our data indicated that MDA levels in lung homogenate of diabetic rats treated with the high concentration were significantly lower than the low concentration (*P* < 0.01) (Table 2). Safranal in the diabetic groups inhibited the level of MDA in BALF supernatant and lung homogenate. The median effective dose (ED_50_) values were 0.42 and 0.48 mg/kg, respectively.

There was a decrease in SOD and CAT in the STZ-diabetic group compared with respective control group (*P* < 0.001). The safranal concentrations (0.25, 0.5, and 0.75 mg/kg/day) significantly increased in SOD activity among the diabetic-treated groups compared with the diabetic group (*P* < 0.001). The CAT activity in diabetic safranal (0.25, 0.5, and 0.75 mg/kg/day)—treated groups, was significantly higher than nondiabetic group (*P* < 0.05, *P* < 0.001). So that, there was not a significant difference between diabetic safranal (0.75 mg/kg/day)—treated groups and control group (Tables 1 and 2).

CAT activity in BALF of animals that have received the high and middle safranal concentrations was significantly greater than that with the low concentration (*P* < 0.05, *P* < 0.001), (Table 1) and that of high concentration inlung homogenate was significantly greater than the low concentration (*P* < 0.01) (Table 2). In addition, the SOD activity of BALF and lung homogenate of animals that have been administrated the high safranal concentration was significantly greater than the low concentration (*P* < 0.05, *P* < 0.001) (Table 1) as well as in lung homogenate was higher than the middle concentration (*P* < 0.01), (Table 2). Safranal in the diabetic groups increased the activity of CAT and SOD in BALF supernatant and lung homogenate. The ED_50_ values were 0.33, 0.26 in BALF and 0.35, 0.46 mg/kg in lung, respectively.

STZ injection produced significant increase of NO compared to control group (*P* < 0.001). The safranal concentrations (0.25. 0.5, and 0.75 mg/kg/day) significantly decrease NO level in BALF and lung homogenate in diabetic-treated groups compared with the nontraeted diabetic group (*P* < 0.001) (Tables 1 and 2). NO levels in BALF and lung homogenate of animals that have received the high safranal concentration were significantly lower than the low and medium concentrations (0.25 and 0.5 mg/kg/day), and those of medium were lower than the low concentration (*P* < 0.001) (Tables 1 and 2). Safranal in the diabetic groups inhibited the level of NO in BALF supernatant and lung homogenate. The ED_50_ values were 0.58 and 0.71 mg/kg, respectively.

## 4. Discussion

The results of the present study indicate that intraperitoneal injection of safranal significantly ameliorated increased biomarkers of oxidative stress in rats' lung after STZ administration. We observed the significant elevation in GSH, CAT, and SOD with reduction in the MDA and NO, in both BALF and lung of safranal treated diabetic rats compared with non-treated diabetic group. These results are compatible with the findings reported by other investigations using saffron and its active constituents, crocin, and safranal to improve oxidative damage due to STZ and alloxan diabetic rats [21–24]. In the present study, significant decline in GSH level and antioxidant enzymes activity including SOD, CAT in BALF supernatant and in lung homogenates of STZ diabetic rats in relation to the controls group as well as increased MDA and NO indicates that the increase of oxidative stress and possible damages to the lung structure caused by DM. These data are in accordance with the findings of other authors, [25] who demonstrated the increase of the oxidative stress and the decrease of the antioxidant enzyme SOD in the lungs of diabetic rats. An increase in the expression of inducible nitric oxide synthase in the lung tissue of the diabetic animals has been also indicated by these authors. The same finding was demonstrated by another group of authors [26]. However, they used, as an experimental model, alloxan-induced DM in rabbits. One of the factors responsible for pulmonary alterations can be oxidative stress. The mechanism responsible for this development is hyperglycemia, which activates the polyol pathway, increasing the production of sorbitol. This increase results in cellular stress that leads to a decrease in the intracellular antioxidant defenses. It can also result in the concentration of the products of advanced glycosylation, thus altering cell function. However, hyperglycemia can also activate nuclear transcription factors, triggering an increase in the expression of the inflammatory mediators. The combination of these mechanisms alters the production of oxidants, causing cellular stress and consequently the structural damage [27]. Several studies showed that STZ produces imbalance between plasma oxidant and antioxidant content results in the development of DM and its complications. STZ enters the ß cell via the low affinity glucose protein-2 transporter, inducing the selective destruction of the insulin producing islets' ß cells and, in turn, a drastic reduction in insulin production. The cytotoxic effect of STZ could result from the combined action of DNA alkylation [27] and the cytotoxic effects of ROS [28] or the intracellular liberation of NO directly or indirectly through the formation of peroxynitrite [29, 30].

The improvement of variable measurements in the BALF and lung homogenate of STZ-diabetic rats after safranal treatment might suggest a protective influence of safranal against STZ action on lung tissue damage that might be mediated through suppression of oxygen free radicals induced by STZ. Safranal, monoterpene aldehyde, which is the major constituent of the essential oil of saffron showed good antioxidant activity.

Treatment with safranal reversed diabetic effects on lung GSH level and SOD and CAT activity. Treatment with safranal also decreased MDA and NO in lung of diabetic rats. These results indicate that safranal therapy may reverse diabetic oxidative stress in an overall sense.

Safranal induced an increase in cellular GSH content which might enhance the GSH/GSSG ratio and decrease lipid peroxidation, therefore, improve glucose regulation. In addition, SOD is responsible for removal of superoxide radicals and catalase decomposes hydrogen peroxide to water and oxygen; thus, these enzymes may contribute to the modulation of redox state of lung [31]. This observation perfectly agrees with those of Rahbani et al. 2011 who demonstrated hypoglycemic and antioxidant activity of ethanolic saffron in streptozotocin induced diabetic rats [24]. Similarly, Kianbakht and Hajiaghaee 2011 observed that saffron, crocin, and safranal may effectively control glycemia in the alloxan induced diabetes model of the rat [22]. Furthermore, Kianbakht and Mozaffari, in 2009 indicated that saffron, crocin, and safranal may prevent the gastric mucosa damage due to their antioxidant properties by increasing the glutathione levels and diminishing the lipid peroxidation in the rat gastric mucosa. These studies indicated that safranal was a potent antioxidant and able to protect body organs against certain toxic materials [21].

In conclusion, findings of the present study show that safranal treatment may be effective to prevent lung damage in diabetic rat by modulation oxidative stress. These finding supports the efficacy of safranal as natural antioxidant for diabetes and its complication management.

## Figures and Tables

**Table 1:** GSH, MDA, SOD, CAT, and NO in BALF of control (C), diabetic (D), diabetic + (0.25 mg/kg/day) safranal-treated (D + S1), diabetic + (0.5 mg/kg/day) safranal-treated (D + S2), and diabetic + (0.75 mg/kg/day) safranal-treated (D + S3) rats following 4 weeks of study.

BALF	C	D	D + S1	D + S2	D + S3
MDA (nmol/mg protein)	0.74 ± 0.10	2.08 ± 0.12***	1.68 ± 0.10***	1.52 ± 0.15^∗∗,+^	0.88 ± 0.13^+++,##,×^
GSH (nmol/mg protein)	2.38 ± 0.21	0.46 ± 0.10***	1.36 ± 0.12^∗∗,++^	1.82 ± 0.12^+++^	2.32 ± 0.17^+++,##^
SOD (U/mg protein)	4.70 ± 0.51	1.54 ± 0.27***	3.26 ± 0.30^∗∗,+^	4.00 ± 0.30^∗,+++^	4.80 ± 0.25^+++,#^
CAT (U/mg protein)	2.24 ± 0.20	0.44 ± 0.12***	1.12 ± 0.12^∗∗∗,+^	1.84 ± 0.13^+++,#^	2.14 ± 0.11^+++,###^
NO (*μ*mol/L)	1.98 ± 0.71	13.08 ± 1.08***	10.02 ± 0.71^∗∗∗,+^	6.80 ± 0.64^∗,+++,#^	3.00 ± 0.57^+++,###,×^

Each measurement has been done at least in triplicate and the values are the means ± SEM for eight rats in each group.

Statistical significance for the difference between the data of control versus other groups: *P < 0.05, **P < 0.01, ***P < 0.001.

Statistical significance for the difference between the data of diabetes versus treated groups: ^++^ *P* < 0.01, ^++^ *P* < 0.01, ^+++^ *P* < 0.001.

Statistical significance for the difference between the data D + S1 versus D + S2 and D + S3 (^#^ *P* < 0.05,^##^ *P* < 0.01, ^###^ *P* < 0.001).

Statistical significance for the difference between the data between D + S2 versus D + S3 (^×^ *P* < 0.05).

**Table 2:** GSH, MDA, SOD, CAT, and NO in lung homogenate of control (C), diabetic (D), diabetic + (0.25 mg/kg/day) safranal-treated (D + S1), diabetic + (0.5 mg/kg/day) safranal-treated (D + S2), and diabetic + (0.75 mg/kg/day) safranal-treated (D + S3) rats following 4 weeks of study.

Lung	C	D	D + S1	D + S2	D + S3
MDA (nmol/mg protein)	4.12 ± 0.86	14.61 ± 0.88***	11.56 ± 0.54^∗∗∗,+^	9.50 ± 0.48^∗,+++^	7.92 ± 0.36^+++,##^
GSH (nmol/mg protein)	21.60 ± 0.92	13.88 ± 0.28***	17.24 ± 0.40^∗∗∗,+++^	18.74 ± 0.25^∗∗,+++^	21.16 ± 0.48^+++,###,×^
SOD (U/mg protein)	8.40 ± 0.43	2.00 ± 0.35***	4.00 ± 0.31^∗∗∗,++^	5.40 ± 0.43^∗∗∗,+++^	7.88 ± 0.26^+++,###,××^
CAT (U/mg protein)	6.16 ± 0.35	2.84 ± 0.28***	4.12 ± 0.22^∗∗∗,+^	5.11 ± 0.26^+++^	5.68 ± 0.28^+++,##^
NO (*μ*mol/L)	19.41 ± 3.88	86.00 ± 1.70***	71.00 ± 2.91^∗∗∗,+^	49.61 ± 3.32^∗∗∗,+++,###^	30.41 ± 3.23^+++,###,××^

Each measurement has been done at least in triplicate and the values are the means ± SEM for eight rats in each group.

Statistical significance for the difference between the data of control versus other groups: *P < 0.05, **P < 0.01, ***P < 0.001.

Statistical significance for the difference between the data of diabetes versus treated groups: ^+^ *P* < 0.05, ^++^ *P* < 0.01, ^+++^ *P* < 0.001.

Statistical significance for the difference between the data D + S1 versus D + S2 and D + S3 (^##^ *P* < 0.01, ^###^ *P* < 0.001).

Statistical significance for the difference between the data between D + S2 versus D + S3 (^×^ *P* < 0.05, ^××^ *P* < 0.01).
