# Corrigendum to “Evaluation of Lung and Bronchoalveolar Lavage Fluid Oxidative Stress Indices for Assessing the Preventing Effects of Safranal on Respiratory Distress in Diabetic Rats”

**DOI:** 10.1155/2020/6452878

**Published:** 2020-12-21

**Authors:** The Scientific World Journal

The article titled “Evaluation of Lung and Bronchoalveolar Lavage Fluid Oxidative Stress Indices for Assessing the Preventing Effects of Safranal on Respiratory Distress in Diabetic Rats” [1] was found to contain material from an article by the same authors that was not cited [2], which also studied the effects of safranal in a rat model of diabetes. The article is as follows:

Saeed Samarghandian, Abasalt Borji, Mohammad Bagher Delkhosh, and Fariborz Samini, “Safranal treatment improves hyperglycemia, hyperlipidemia and oxidative stress in streptozotocin-induced diabetic rats,” *Journal of Pharmacy and Pharmaceutical Sciences* (2013), 16 (2). pp. 352–362.

The authors do not agree to the publication of this corrigendum. In the previous article [2], blood was drawn for assaying the biochemical parameters in order to determine the changes to cellular antioxidant defence systems and antioxidant enzymes, while in this article [1], the therapeutic effect of safranal against lung oxidative damage was assessed and there is no overlap in results.
